# Harnessing tumorous flaws for immune supremacy: is miRNA-155 the weak link in breast cancer progression?

**DOI:** 10.1172/JCI163010

**Published:** 2022-10-03

**Authors:** Samantha Sharma, Mateusz Opyrchal, Xiongbin Lu

**Affiliations:** 1 Department of Medical and Molecular Genetics and; 2 Department of Medicine, Division of Hematology/Oncology, Indiana University School of Medicine, Indianapolis, Indiana, USA.; 3 Indiana University Melvin and Bren Simon Comprehensive Cancer Center, Indianapolis, Indiana, USA.

## Abstract

With the advent of immune checkpoint blockade (ICB) therapy, treatment strategies for late-stage cancers have seen a radical advancement. In this issue of the *JCI*, Wang et al. characterize the functional role of miR-155 in breast cancer and its potential in harnessing the efficacy of immunotherapy. The study reports that high expression levels of miR-155 in breast cancer cells downregulated suppressor of cytokine signaling 1 (SOCS1), increased the phosphorylated STAT1 (pSTAT1)/pSTAT3 ratio, and thereby stimulated chemoattractants for tumor infiltration of effector T cells. Moreover, miR-155 overexpression set the stage for ICB therapy via increased programmed death ligand 1 (PD-L1) expression on cancer cells and enhanced immunological memory response via the release of miR-155–containing extracellular vesicles. Collectively, these data suggest that miR-155 is a strong candidate as a prognostic biomarker for ICB therapy.

## miRNAs at the crossroads between inflammation and cancer

For decades, researchers have speculated that inflammation and cancer are like a fighting pair in a relationship (1). Further igniting the mystery, miRNAs have emerged as a ringleader for this duo (2). A class of small noncoding RNAs, known as microRNAs (miRNAs), play an essential role in controlling cellular processes by regulating their target gene expression (3). Dysregulation of miRNAs contributes to the development of several inflammatory diseases as well as cancer (4, 5). On the one hand, miRNAs are upregulated by a myriad of inflammatory mediators and, in turn, modulate inflammatory responses by fine-tuning target gene expression. On the other hand, miRNAs can act oncogenically, in which case they are termed oncomiRs, promoting cancer initiation and progression in a variety of tissues. Both inflammation and carcinogenesis hijack the immune system, and the recent reports highlighting the role of miRNAs in the modulation of immune cell functions are the frosting on the cake (so to speak) (6).

The crosstalk among miRNAs, inflammation, carcinogenesis, and immunity is complex and not yet fully understood. Recent findings in different cancer types have established miRNAs as promising biomarkers for diagnosis, prognosis, and response to therapies (5). However, a critical question remains: Can we utilize the miRNAs to modulate immune function to address diseases affecting humans? To answer this question, Wang et al., in this issue of the *JCI*, have attempted to understand the antitumor role of one such oncomiR, miR-155, in breast cancer and its potential in harnessing the efficacy of immune checkpoint blockade (ICB) therapy (7).

## Multifaceted roles of miR-155

A large-scale miRNome analysis revealed that miR-17-5p, miR-20a, miR-21, miR-92, miR-106a, and miR-155 are the top-ranked candidates for cancer pathogenesis (8). Among those pathological miRNAs, miR-155 has emerged as one of the key miRNAs in large cell lymphoma, Burkitt lymphoma, various B cell lymphomas, breast cancer, lung cancer, and colon cancer. Recent studies have also identified a secondary role of miR-155 in the immune-enriched microenvironment in 30 tumor types, in which it acts by stimulating immunosuppressive myeloid-derived suppressor cells and immunocompetent DCs (9). Primary miR-155 is transcribed from exon 3 of the B cell integration cluster (BIC; or the host gene MIRHG155) located on chromosome 21. Following nuclear and cytoplasmic processing, pre-miR-155 is converted into a 22-nucleotide miR-155 duplex containing -5p and -3p strands. Despite an identical biogenesis precursor, miR-155-5p and miR-155-3p are like epigenetic twins, presenting diverse and occasionally antagonistic functions due to alternative cleavage and polyadenylation.

Early studies first identified miR-155 as an oncomiR in breast cancer (8). In breast cancer, miR-155 is differentially expressed in estrogen receptor–negative (ER-negative) versus ER-positive tumors (10). However, in-depth analyses of The Cancer Genome Atlas (TCGA) data sets by Ekiz et al. argue that it may not be the case (9). In this study, the authors found that miR-155 was not only upregulated in specific cancer types, but was also associated with improved overall survival of patients with melanoma, breast, and colon cancer (9). The marked knowledge gap in elucidating the role of miR-155 in breast cancer can be attributed to the in vitro models or immunodeficient animal models that lack fully functioning immune components. The syngeneic mouse models used by Wang et al. overcome these limitations and provide an extensive understanding of the biological functions of miR-155 in breast cancer (7).

**Figure 1:**
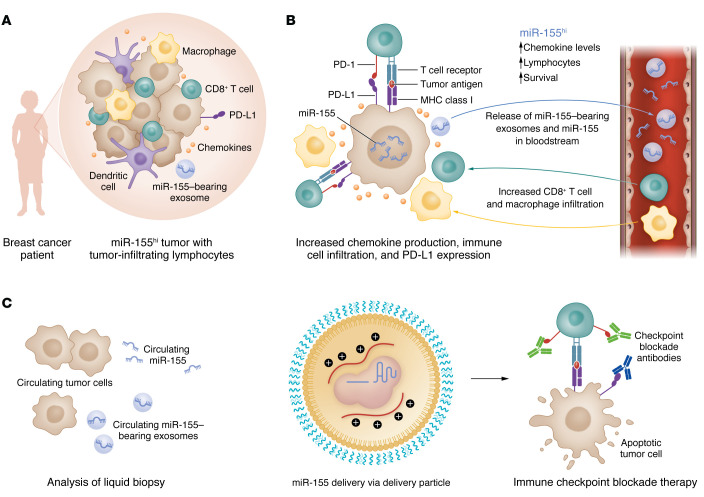
The role of miR-155 in breast cancer and its therapeutic translational potential. (**A**) Breast cancer patients with an elevated level of miR-155 (miR-155^hi^) have increased levels of tumor-infiltrating lymphocytes. (**B**) Mechanistically, tumor-associated miR-155 can upregulate chemokine production, specifically, CXCL9, CXCL10, CXCL11, and PD-L1 expression on the tumor cells. Furthermore, tumors expressing elevated levels of miR-155 release miR-155–enriched exosomes in the bloodstream. Overall, miR-155^hi^ breast tumors have a better overall patient survival rate, elevated chemokine production, and an increase in tumor-infiltrating lymphocytes as compared with miR-155^lo^ breast tumors. (**C**) At the clinical level, miR-155 and miR-155–enriched exosomes in liquid biopsy of patients can be used as positive prognostic markers for breast cancer. Further, therapeutic delivery of miR-155 may improve the effectiveness of ICB therapy via increased PD-L1 expression.

Utilizing publicly available TCGA data, Wang et al. (7) confirmed that miR-155 levels are upregulated in breast cancer patients and positively correlated with overall patient survival (Figure 1). Moreover, patients with stage I/II breast cancer and those without lymph node involvement had higher miR-155 levels than patients with advanced cancer stages and those with lymph node metastasis, respectively. To validate the function of tumor-derived miR-155 in tumor growth, the authors established miR-155–overexpressing tumors in miR-155–knockout mice. Interestingly, the elevated miR-155 levels in the tumor cells were sufficient to suppress tumor progression and metastasis, and miR-155 depletion in the tumor cells reversed the antitumor effects. These data indicated that miR-155 is important in regulating tumor progression, and its overexpression results in decreased tumor progression and suppression of metastasis (7). However, due to the lack of single-cell RNA-Seq data for human breast cancer tissues, it is unknown what the primary source of miR-155 is, whether there is altered miR-155 expression in tumor-associated stromal cells (fibroblasts, immune cells, and endothelial cells), and how it contributes to tumorigenesis in human breast cancer.

## miR-155 is tumor derived but immune favored

In recent reports, aberrant expression of miR-155 was observed in a variety of activated immune cells, leading to impaired immune functions (11, 12). As an example, a study on the expression of miR-155 in four types of immune cells — DCs, B cells, CD4^+^ T cells, and macrophages — provides evidence of the cellular context–specific role of miR-155 in gene regulation (13). In plasmacytoid DCs, miR-155-3p upregulates IFN-α/β, while miR-155-5p downregulates IFN-α/β (14). In the ovarian cancer model, miR-155 switched the immunosuppressive tumor-associated DCs to an immunostimulatory phenotype (15). Given its wide spectrum expression in immune cells, investigation of miR-155 in tumor-immune crosstalk is warranted.

Based on the in silico analysis performed on differential RNA-Seq data analysis, Wang et al. found that patients with higher miR-155 levels consistently had upregulated genes spanning antitumor and immune response pathways. These pathways included lymphocyte activation and interferon signaling and correlated with upregulation of the expression of proteins associated with T cell activity. Furthermore, in their in vivo mouse models, Wang et al. elucidated the role of tumor-derived miR-155 in attracting CD45^+^ immune cells. As a result, elevated levels of miR-155 increased the presence of DCs, helper T cells, cytotoxic T cells, and natural killer cells. Compared with the control counterparts, higher expression levels of miR-155 downregulated suppressor of cytokine signaling 1 (SOCS1), increased the ratio of phosphorylated STAT1 (pSTAT1) to pSTAT3, and stimulated chemoattractants CXCL9, CXCL10, and CXCL11 for T cell activation and subsequent proimmune antitumor response. In human breast cancer, miR-155 levels were positively correlated with the presence of CD8^+^ T cells and M1 macrophages in the tumor microenvironment (TME), making it a fair case for exploring the importance of miR-155 in cancer immune regulation and its potential in cancer immunotherapy (7).

## Is miR-155 a friend or foe?

Despite the original identification of miR-155 as an oncomiR, the correlation of miR-155 with better clinical outcomes in cancer patients points to a different angle of this story. It is intriguing to see that upregulation of miR-155 can positively or negatively affect the expression of immune checkpoints in certain cancer types (16, 17). To explore this aspect of miR-155 in breast cancer, Wang et al. studied the effect of miR-155 on immune checkpoints in both tumor and immune cells. They found that miR-155 overexpression increased CD274 (programmed death ligand 1 [PD-L1]) expression on cancer cells and tumor-associated macrophages (Figure 1) (7). With the discovery of immune checkpoint inhibitors, increased PD-L1 expression has been proposed as a predictive marker for increased responses (18), which makes miR-155 a suitable candidate for exploration in breast cancer immunotherapy. Using their in vivo breast cancer model, Wang et al. showed that miR-155 overexpression sensitized tumors to the treatment of PD-L1 monoclonal antibodies. Overall, miR-155 overexpression in the breast cancer model not only increased tumor-infiltrating lymphocytes and decreased tumor growth, but also set the stage for effective ICB therapy via increased PD-L1 expression (7).

## Extracellular vesicles as cargo carriers and tumor-immune communicators

The TME consists of tumor cells, the extracellular matrix, immune cells, blood vessels, and other cell types, including fibroblasts that are likely to determine the course of the disease. The cellular and cell-matrix interactions within the TME are highly complex. In the last decade, we have seen substantial growth in understanding the role of TME-derived extracellular vesicles (EVs), such as exosomes, for cell migration and cell-to-cell communication in cancer metastasis (19). These EVs are known to carry a variety of biomolecules, including miRNAs, and have earned the reputation of acting as cancer cargos. In the present study, Wang et al. confirmed the presence of miR-155–enriched exosomes in the TME of breast cancer (Figure 1). Furthermore, administration of tumor-derived exosomes augmented the immune response in mammary tumor–bearing mice and established immunological memory, as confirmed by tumor rechallenge experiments. Intriguingly, the authors found that in breast tumor mouse models, intravenous administration of exosomes derived from miR-155–overexpressing tumors augmented the immune response, as characterized by increased CD45^+^ immune cells and cytotoxic CD8^+^ T cells in tumor tissues. At the clinical level, the expression levels of miR-155 in the serum of breast cancer patients correlated with the miR-155 levels in the breast tumor tissue, indicating miR-155 is a potential prognostic marker for breast cancer (7).

## Translational implications of miR-155

Early predication and timely treatment are vital to improving the clinical outcome of cancer patients. Understanding the complexity and specific biomarkers for each cancer type and subtype is pertinent to recommending the most effective treatments for patients. Based on the available categorization of mutational burden, the BRCAness or BRCA1/2 mutation load confers human breast cancer responsiveness to poly (adenosine diphosphate-ribose) polymerase (PARP) inhibitors. Similarly, high expression of PD-L1 predicts a greater success rate in patients with metastatic breast cancer. Following these guidelines, could EVs or nanomedicine delivery for miRNAs do the magic?

Liquid biopsy is an essential noninvasive approach to deciphering the mutational burden, genomic makeup, and tumor biomarkers in circulation and can be used for continual monitoring of cancer and its response to therapies. Wang et al. have shown relevant data to propose higher levels of miR-155 expression and miR-155–bearing exosomes as a positive prognostic biomarker for breast cancer. Based on the miR-155 levels, patients can be provided tailored treatments and a combination of drugs with ICB therapy with the potential to deescalate nontargeted chemotherapy, thus reducing toxicity.

Cancer immunotherapy has revolutionized the field of cancer treatment; however, we are a long way from having an exceptional success rate. Recent advances in single-cell genomics have greatly added to unveiling the dysregulated pathways and genes. Biomarkers predictive of immune checkpoint inhibitor efficacy and resistance are needed to select the best treatment for patients and develop new treatment strategies for patients whose disease progressed after immune checkpoint inhibitor monotherapy. Despite recent successes of single-agent PD-1 blockade for mismatch repair–deficient, locally advanced rectal cancer (20) and improved survival in pancreatic cancer patients treated with PD-1 blockade and chemotherapy (21), most patients with solid malignancies receive limited or no benefit from these approaches. Undoubtedly, it will be of great interest to determine whether miR-155 levels in liquid biopsy can be used as a prognostic biomarker alone or with PD-L1 status for breast cancer ICB therapy.

## Remaining questions to consider

What are the next steps toward utilizing miRNAs for cancer treatment? First, we suggest deepening our understanding of the role of miRNAs and their relation to inflammation and metabolism and how this information can mold miRNAs for effective therapeutics. Answering the following questions will be helpful: (a) Beyond higher expression, how is the transcriptional regulation and/or posttranscriptional processing of miR-155-5p and miR155-3p modulated in cancer? (b) How does miR-155 modulate cancer metabolism in breast cancer patients and affect the tumor-infiltrated immune cell populations? (c) How do inflammation and miR-155 evolve as the disease progresses, from the early to late stages of breast cancer? What is the status and function of the miR-155 expression level in different subtypes of breast cancer? (d) What precludes the cancer killing in early breast cancer stages even though the tumor tissue has higher levels of miR-155? (e) Is there a therapeutic value to using miR-155 as a small RNA drug for breast cancer therapy?

Despite rapid development of RNA biology and technology in cancer research, RNA-based therapeutics, including those that use small-interfering RNAs, miRNAs, antisense oligonucleotides, and synthetic mRNAs, need to overcome the barriers in specificity, stability, and delivery before these RNA modalities reach their full potential. However, as the proverbial saying goes, “Every dark cloud has a silver lining.” We believe that the success of COVID-19 mRNA vaccines will greatly accelerate research on RNA-based therapeutics in human cancer. It is anticipated that miRNAs may transpire as one of the most effective weapons against cancer.

## Version 1 10/03/2022

Electronic publication
